# Maladie de hodgkin et cancer colique secondaire: à propos d'un cas

**DOI:** 10.4314/pamj.v9i1.71200

**Published:** 2011-07-06

**Authors:** Lamia Boulaâmane, El Mehdi Tazi, Meryem Glaoui, Ghizlane Raiss, Issam Lalya, Saber Boutayeb, Hind M'rabti, Hassan Errihani

**Affiliations:** 1 Service d'Oncologie Médicale, Institut National d'Oncologie, code postal 10100, Rabat, Maroc; 2 Service de radiothérapie, Institut National d'Oncologie, code postal 10100, Rabat, Maroc

**Keywords:** Maladie de Hodgkin, cancers secondaires, adolescents survivants, radiothérapie, radio-chimiothérapie, chimiothérapie

## Abstract

La maladie de HODGKIN (MDH) survient le plus souvent chez les enfants, adolescents et jeunes adultes. Elle représente une des tumeurs malignes les plus curables. Grâce aux progrès thérapeutiques actuels, plusieurs patients ont pu obtenir des réponses complètes durables, mais les tumeurs solides malignes, secondaires au traitement, demeurent la complication tardive la plus redoutable chez les longs survivants. Il s'agit d'un patient de 28 ans, sans antécédents pathologiques notables, suivi depuis l'age de 13 ans pour une MDH scléro-nodulaire stade IV (à localisation pulmonaire). Le patient a reçu initialement 06 cures de chimiothérapie, suivies d'une radiothérapie externe sus et sous-diaphragmatique. L’évolution a été marquée par la survenue de 02 rechutes, pour lesquelles, 02 autres lignes thérapeutiques ont été administrées. Puis, survenue d'une progression, pour laquelle il a été mis sous Cyclophosphamide par voie orale. Après 02 ans d'un bon contrôle clinique et radiologique sous cette dernière drogue cytotoxique, le patient a fait apparaître une hématurie macroscopique et parallèlement, un adénocarcinome (ADK) colique, survenant 15 ans après le début du traitement initial du lymphome, ayant nécessité une hémicolectomie droite élargie. Les modalités thérapeutiques de la MDH chez les adolescents et jeunes adultes ont subit des modifications remarquables ces dernières décennies. L’évaluation du risque de cancers secondaires chez les longs survivants, reflète souvent l'effet de modalités anciennes plus agressives. La plupart des études rapportant des cas de cancers secondaires, tiennent en compte la 1^ère^ ligne thérapeutique et les traitements de rattrapage de la MDH. En effet, les jeunes patients traités par chimiothérapie et plus particulièrement par radiothérapie, sont à haut risque de développer des cancers secondaires. La radio-chimiothérapie parait par ailleurs, augmenter de façon plus significative ce risque. L'estimation du risque de cancers secondaires à long terme demeure alors essentielle, afin de minimiser les complications tardives, et ceci à travers des mesures de prévention et de dépistage chez les jeunes patients potentiellement à risque.

## Introduction

Le taux de survie des enfants et adolescents suivis pour une maladie de Hodgkin (MDH) atteint approximativement 90% après 10 ans de traitement [1]. Mais, en dépit des progrès thérapeutiques réalisés ces 30 dernières années, les jeunes patients recevant précocement des traitements plus agressifs demeurent à risque de complications tardives, notamment les cancers secondaires.

En effet, un excès de leucémies aigues chez les patients traités par chimiothérapie et une augmentation du risque de tumeurs solides chez les patients traités par radiothérapie, ont été longtemps rapportés dans la littérature.

La détermination du risque absolu (RA), lié à l’âge, au sexe, et à la prédisposition génétique aux cancers secondaires chez les longs survivants de la MDH, a une importance capitale. Elle permet une estimation précise du risque individuel, qui demeure toujours plus élevé par rapport à la population générale, et ceci, afin de faciliter le développement de stratégies de dépistage précoce.

## Patient et observation

Il s'agit d'un jeune patient de 28 ans, de sexe masculin, sans antécédents pathologiques notables, suivi depuis l'age de 13 ans pour une MDH scléro-nodulaire stade IV (à localisation pulmonaire bilatérale). Le patient a reçu initialement 06 cures de MOPP (Mechloréthamine et Vincristine à J1 et J8 en intraveineux, Procarbazine et Prédnisone de J1 à J14 par voie orale) tous les 28 jours, suivies d'une radiothérapie externe en sus-diaphragmatique (en mantelet) et en sous-diaphragmatique (en Y inversé) à la dose de 20Gy: 2Gy par fraction, en 10 séances.

Six ans après un bon contrôle loco-régional, le patient a présenté une rechute ganglionnaire, pour laquelle il a été mis sous chimiothérapie de 2^ème^ ligne type ABV (Adriamycine, Vinblastine, Bléomycine) à J1 et J14, en intraveineux, tous les 28 jours, avec une réponse partielle après 06 cures. Une 2^ème^ rechute est survenue après un délai de 07 ans, nécessitant l'administration d'une 3^ème^ ligne de chimiothérapie type Etoposide-Cisplatine en intraveineux, tous les 21jours. L’évaluation après 2 cycles de cette dernière a montré une progression radiologique, pour laquelle il a été mis sous Cyclophosphamide par voie orale, avec une stabilisation radiologique et une bonne tolérance clinique pendant 02 ans. Cependant, après cette longue durée de bon contrôle loco-régional, le patient a présenté une hématurie macroscopique de moyenne abondance, nécessitant l'arrêt du Cyclophosphamide, évoluant dans un contexte d'amaigrissement chiffré à 5 Kg en 5 mois. L'examen clinique a retrouvé une masse para-ombilicale gauche de 4cm, dure, fixée au plan profond, sensible, sans adénopathies périphériques et sans hépato-splénomégalie. La cystoscopie a mis en évidence une cystite inflammatoire, et l'imagerie tomodensitométrique a montré un épaississement digestif de 3cm/6,5 intéressant le colon transverse. La biopsie de ce dernier a révélé un adénocarcinome (ADK) moyennement différencié de type lieberkuhnien. Une hémicolectomie droite élargie a été réalisée, l'examen anatomopathologique était en faveur d'un ADK colique, classé pT3N0 (13 ganglions prélevés non envahis), à limites de résection saines, sans embols vasculaires, ni engainement périnerveux. Le patient est actuellement en bon contrôle clinique et radiologique.

## Discussion

La survenue de cancers secondaires après traitement de la MDH est une notion reconnue depuis 1970. La plupart des études rapportent un risque accru de leucémies, secondaires à l'usage d'agents alkylants. Plus récemment, les lymphomes non hodgkiniens (LNH) et les tumeurs solides deviennent plus fréquents après radiothérapie, en particulier, le cancer du sein, le cancer de la thyroïde et les tumeurs gastro-intestinales malignes.

Contrairement aux leucémies, l'incidence des tumeurs solides n'est pas en plateau après 10 ans de traitement, elle est plutôt en croissance continue avec le temps.

Plusieurs études ont déjà montré que, contrairement aux leucémies, le risque relatif (RR) de tumeurs solides augmente progressivement au cours du suivi, en moyenne; 5 à 20 ans après le traitement initial de la MDH [2]. Par ailleurs, aucune étude n'a pu estimer le RA de survenue de cancers secondaires au cours d'un suivi prolongé, en fonction de plusieurs variables; notamment, l'age au diagnostic de la MDH et l'age atteint, le temps écoulé depuis le diagnostic, le sexe et le type du traitement initial.

Le risque des tumeurs solides à un site particulier, en fonction de l'age du diagnostic de la MDH a été abordé dans plusieurs études, mais peu d'entre elles, a pu le déterminer chez les patients traités particulièrement avant l'age de 20 ans. Néanmoins, une étude rapporte une survenue élevée de cancer de la thyroïde et du tractus respiratoire (poumon et plèvre), chez des enfants traités avant l'age de 10 ans. Entre 10 et 16 ans, le siège prédominant des cancers secondaires était celui du tractus digestif et du sein [3].

Concernant les tumeurs digestives malignes plus particulièrement, plusieurs études récentes ont montré différemment, que les patients traités pour une MDH avant l'age de 25 ans, ont un risque plus élevé de survenue de cancer colorectal, sans excès de ce risque chez les patients âgés de plus de 45 ans [4]. Et que le RR de sa survenue avant l'age de 40 ans est significativement plus élevé qu'entre 40 et 49 [2], survenant 10 à 25 ans avant l'age o[ugrave] le dépistage de routine est recommandé pour la population générale.

L'influence de la chimiothérapie dans la survenue de cancers secondaires varie selon la localisation de ces derniers. En effet, il est démontré que le risque de survenue des tumeurs solides après chimiothérapie de rattrapage lors d'une rechute est plus marqué pour les tumeurs gastro-intestinales [2].

Plusieurs études rapportent un risque élevé de survenue de tumeurs solides chez des patients traités par chimiothérapie pour une MDH, notamment par des agents alkylants, des anthracyclines, et des épipodophyllotoxines. Néanmoins, le rôle de ces drogues cytotoxiques dans leur pathogénie n'est pas très bien établi, contrairement à la radiothérapie.

En se basant sur les résultats d’études menées chez les survivants de la bombe atomique, qui montrent que les jeunes adultes sont particulièrement susceptibles aux effets carcinogènes de l'irradiation [5], d'autres études rapportent un excès du risque de tumeurs malignes du tractus digestif, particulièrement le cancer gastrique, à un âge précoce d'exposition [6]. Les enfants et adolescents, sont également à plus haut risque de développer le cancer de l’œsophage et du rectum par rapport aux adultes [7].

Selon la littérature, le cancer du sein représente la tumeur solide la plus fréquente, qui a été rapportée chez les jeunes patientes survivantes, traitées par radiothérapie en mantelet pour une MDH, mais, a été également décrit chez les hommes [8]. D'autres cancers sont également liés à un effet tardif de la radiothérapie, tels que le cancer de la thyroïde, l'ostéosarcome, les sarcomes des tissus mous et le cancer du poumon [9]. Cependant, il a été démontré que les patients recevant une chimiothérapie de rattrapage après une radiothérapie initiale, ont un risque significativement plus élevé de développer des tumeurs solides autres que le cancer du sein, par rapport aux patients recevant uniquement une radiothérapie [2]. Ce risque est encore plus significatif avec l'association radio-chimiothérapie [10]. Ceci reflète vraisemblablement le fait que, les patients qui rechutent après radiothérapie et reçoivent une chimiothérapie de rattrapage, sont classés dans le groupe de patients recevant une radio-chimiothérapie dans la plupart des études antérieures.

## Conclusion

Malgré que les nouvelles approches thérapeutiques tentent d'optimiser les taux de guérison des jeunes patients traités pour une MDH, il est important de réduire les complications précoces et tardives, tout en limitant les doses et les champs d'irradiation. Il importe également de définir plus clairement le rôle de la chimiothérapie dans la pathogénie des tumeurs malignes secondaires, afin de réduire la morbi-mortalité liée à ces cancers. D'autres études s'avèrent encore nécessaires, dans le but de déterminer les caractéristiques des patients considérés spécialement à haut risque, quant à leur exposition aux radiations ionisantes et aux agents cytotoxiques, et surtout d'explorer les altérations génétiques provoquées par les différents types de traitement, plus particulièrement induits par la radiothérapie.
